# Combined Fruit and Vegetable Intake Is Correlated with Improved Inflammatory and Oxidant Status from a Cross-Sectional Study in a Community Setting

**DOI:** 10.3390/nu4010029

**Published:** 2012-01-04

**Authors:** Martin M. Root, Megan C. McGinn, David C. Nieman, Dru A. Henson, Serena A. Heinz, R. Andrew Shanely, Amy M. Knab, Fuxia Jin

**Affiliations:** 1 Department of Nutrition and Health Care Management, Appalachian State University, ASU Box 32168, Boone, NC 28608, USA; 2 Silver Bluff Village, 100 Silver Bluff Drive, Canton, NC 28716, USA; Email: mm70477@gmail.com; 3 Human Performance Laboratory, Appalachian State University, North Carolina Research Campus, 600 Laureate Way, Kannapolis, NC 28081, USA; Email: niemandc@appstate.edu (D.C.N.); shanelyra@appstate.edu (R.A.S.); knabam@appstate.edu (A.M.K.); 4 Department of Biology, Appalachian State University, Boone, NC 28608, USA; Email: hensonda@appstate.edu; 5 Carver College of Medicine, University of Iowa, 2206 Medical Education & Research Facility, Iowa City, IA 52242, USA; Email: serena-heinz@uiowa.edu; 6 Dole Nutrition Research Laboratory, 600 Laureate Way, Kannapolis, NC 28081, USA; Email: fuxia.jin@dole.com

**Keywords:** fruits, vegetables, inflammation, oxidant status

## Abstract

Previous studies have examined the relationship between specific nutrient and food intakes with limited markers of either inflammation or oxidant status. The objective of this study was to determine if an increase in combined self-reported fruit and vegetable (F&V) intake in a community setting was associated with improved multiple markers of inflammatory and oxidant status. A community group (*N* = 1000, age 18–85 years, 61% female) gave two fasted blood samples separated by 12 weeks. Blood inflammatory biomarkers included total leukocytes (WBC), plasma C-reactive protein (CRP), interleukin-6 (IL-6), IL-10, tumor necrosis factor-alpha (TNF-α), monocyte chemoattractant protein-1, and granulocyte colony stimulating factor. Measured oxidant status markers were ferric reducing ability of plasma (FRAP), oxygen radical absorbance capacity (ORAC) and plasma F_2_-isoprostanes. The relation of markers across categories of F&V intake was examined. In analyses controlling for other important dietary and lifestyle factors, IL-6 and TNF-α were significantly lower across categories of increasing F&V intakes (*p* < 0.008). FRAP and ORAC were significantly higher (*p* < 0.0001 and *p* = 0.047 respectively) while F_2_-isoprostanes was significantly lower (*p* < 0.0001) across F&V categories. In a community study, several markers of both inflammation and oxidant status were associated in a putatively salutary direction by higher intake of combined F&V, supporting current guidelines suggesting increased F&V consumption for the prevention of chronic diseases.

## 1. Introduction

Diets filled with fruits and vegetables (F&V), containing a variety of vitamins, minerals, and antioxidants, have been associated with a lower risk of developing age-related chronic diseases [1]. The beneficial components F&V contain have shown a protective effect against disease-related markers of inflammation and oxidative stress. The World Health Organization noted that inadequate intake of F&V is one of the leading causes of chronic disease and overall death and mortality worldwide [2]. Recent estimates have reported total worldwide mortality currently attributable to inadequate F&V intake is up to 2.635 million deaths per year [1]. Evidence supporting the role of F&V intake in prevention of chronic disease is expanding beyond the role in cancer and heart disease, showing protective effects in the prevention of stroke, cataracts, diverticulitis, diabetes, chronic obstructive pulmonary disease, and hypertension [3]. Because F&V intake is known to decrease the risk of chronic diseases, public health strategies to improve F&V intake should be encouraged.

Circulating markers of oxidative stress and inflammation are known to play a complex role in the development of age-related chronic diseases [4]. Interleukin-6 (IL-6) and C-reactive protein (CRP), markers of systematic inflammation in the body, have been shown to decrease as F&V consumption increases [5,6]. Oxidative stress, a negative balance between free radical oxidation and antioxidants, plays a detrimental role in the development of chronic disease. Plasma and urinary F_2_-isoprostanes, markers of oxidative stress, have also been found to decrease as vegetable intake increases [7,8]. Ferric reducing ability of plasma (FRAP) and oxygen radical absorbance capacity (ORAC), indicators of antioxidant capacity, have been found to increase as F&V consumption increases. Interest is growing relating specific circulating markers of inflammation and oxidative stress with chronic disease and related lifestyle habits.

Studies have examined the relationship between specific nutrients and foods with inflammation and oxidation, but there are few relating combined F&V with both putative mechanistic pathways of chronic disease. The effect of a single food, nutrient, or food group is not always clear; foods and nutrients are consumed in combination and as a result may have a synergistic effect [9]. Analysis of overall dietary patterns provides a comprehensive correlation with their overall effects on oxidation, inflammation, and disease risk. The present study objective was to determine if a self-reported F&V intake was correlated in a putatively salutary direction with markers of both inflammation and oxidative stress while controlling for important confounders.

## 2. Experimental Section

### 2.1. Subjects

Male and female community members were recruited by mass advertising in the local media. Enrolled subjects (*N* = 1023) 18–85 years of age, were studied during 12-week periods from January to April 2008 and from August to November 2008. Women who were pregnant or lactating were not recruited, but no other exclusion criteria were employed. The final number of subjects completing all requirements of the study, including the final blood draw, was 1000. Other survey details were previously described [10]. All study procedures were approved by the Appalachian State University Institutional Review Board, and written informed consent was obtained from each subject. All protocols were in compliance with Health Insurance Portability and Accountability Act (HIPAA) guidelines.

### 2.2. Lifestyle and Clinical Measures

In order to obtain lifestyle habit information, subjects were asked to complete a lifestyle habit survey using an Internet-based site (SurveyMonkey.com, Portland, OR, USA) two weeks prior to the first laboratory visit for the study. A food frequency questionnaire was administered 2 weeks before the first blood draw with subjects asked to check a box representing typical daily consumption of fruits, vegetables, and red meat. For fruit and vegetable intake, this is the same strategy used in the Anti Cancer Council of Victoria Food Frequency Questionnaire (ACCVFFQ) that was validated against a wide number of nutrient intakes [11]. Specifically, questions asked, “On average, how many servings of…do you eat per day?” Serving size information was provided for each food group, and then subjects checked a box representing how many servings they consumed on an average day. For fruit intake the five questionnaire answers were reduced to categories of less than twice daily, twice daily, and greater than twice daily. For vegetable intake the 5 answers were reduced to categories of less than 3 servings, 3 servings, and greater than 3 servings. F&V consumption was aggregated into categories. Due to the nature of the original scales these became the apparently overlapping categories of zero to 2–3 servings, 3–4 to ≥7 servings, and 7 to ≥9 servings. Seventy eight responses were removed from the combined F&V calculations due to unreliable responses. Information on smoking habits was reduced to compare only current smokers and current nonsmokers. Self-reported physical fitness level was assessed on a 10 point Likert scale, with subjects asked to compare their level to other persons of the same age. Other details of the survey are described elsewhere [10]. Height and weight were measured and blood samples were obtained following an overnight fast (between 7 and 9 am) twice separated by 12 weeks. Blood samples were spun and EDTA plasma aliquoted and frozen at −80 °C. Only the complete blood count was measured on fresh blood. These frozen samples were thawed and then analyzed for outcome measures as described below. Unless otherwise specified all chemicals were purchased from Sigma Aldrich (St Louis, MO, USA).

### 2.3. Inflammatory Markers

Enzyme-linked immunosorbant assays (R&D Systems, Inc. Minneapolis, MN, USA) were used to measure total plasma concentrations of interleukin-6 (IL-6, high sensitivity), interleukin-10 (IL-10, high sensitivity), granulocyte colony stimulating factor (GCSF, high sensitivity), monocyte chemoattractant protein-1 (MCP-1), and tumor necrosis factor-α (TNF-α). Serum C-reactive protein (CRP, high sensitivity) was measured using an LX-20 clinical analyzer (Beckman, Brea, Calif., USA). All samples and provided standards were analyzed in duplicate in random order together with standards and standard samples.

### 2.4. Oxidative Status

Plasma F_2_-isoprostanes were determined using gas chromatography-mass spectrometry (GC-MS) [12]. In brief, samples were used to extract free F_2_-isoprostanes together with added deuterated [^2^H_4_] prostaglandin F_2_ as an internal standard. The mixture was then added to a C18 Sep Pak column, followed by silica solid phase extractions. F_2_-isoprostanes were converted to pentafluorobenzyl esters, subjected to thin layer chromatography, and converted to trimethylsilyl ether derivatives. Samples were analyzed by a negative ion chemical ionization GC-MS using an Agilent 6890N gas chromatography interfaced to an Agilent 5975B inert MSD mass spectrometer (Agilent Technologies Inc., Santa Clara, CA, USA) [13]. 

Total plasma antioxidant ability was determined by the ferric reducing ability of plasma (FRAP) assay [14]. In brief, this assay utilizes water soluble antioxidants native to the plasma collected from EDTA treated blood to reduce ferric iron to the ferrous form subsequently producing a chromogen identifiable at 593 nm. Samples and standards are expressed as ascorbate equivalents based on an ascorbate standard curve.

Oxygen radical absorbance capacity (ORAC) was measured using methods described previously [15]. In brief, serial dilutions of Trolox were made using phosphate buffer solution and used as standards. Blanks, trolox standards, and human plasma samples were loaded into appropriate microtiter plate wells, followed by fluorescein working solution. The plate was then incubated with AAPH working solution. ORAC values were calculated by a fluorescence plate reader (Spectra Max Gemini XPS, Molecular Devices) as area under the curve.

### 2.5. Complete Blood Count

A complete blood count (CBC) with leukocyte differential was analyzed in the clinical laboratory of the Watauga Medical Center (Boone, NC, USA) using standard clinical laboratory equipment and quality standards.

### 2.6. Statistical Procedures

Statistical procedures were performed with version 9.2 SAS software (SAS Institute, Cary, NC, USA). Differences in group means were determined by *t*-tests and differences in group percentages by chi-square. Repeated measures generalized linear models were used to determine trend across categories of F&V consumption with post-hoc testing with Bonferroni’s adjustment of differences between categories. Models were adjusted for age and gender or for age, gender BMI, smoking, physical fitness, and red meat intake. Including chronic disease state in the model did not substantially alter the results. Several highly skewed variables were log-transformed before multivariate analysis.

## 3. Results and Discussion

Socio-demographic and clinical characteristics for subjects by gender are presented in Table 1. A total of 1000 subjects (61% women) aged 18–85 completed the study. Subjects were predominately White (95%) and were characterized with more years of education (15.6 years). Thirty-seven percent of subjects (35% men and 39% women) reported past or current history of one or more chronic diseases. Men were generally more physically active and had higher BMIs.

**Table 1:** Subject characteristics by gender (mean ± standard deviation) of community cohort.

Variable, unit of measure	Men	Women	Probability of difference ^a^
	*N* = 394	*N* = 606	
Age, years	45 ± 17	47 ± 16	0.09
Married, %	63%	54%	0.0066
White ethnicity, %	94%	96%	0.16
Chronic disease, %	35%	39%	0.27
BMI, kg/m^2^	27.5 ± 5.0	26.4 ± 5.9	0.0016
Education, years finished	15.6 ± 2.9	15.5 ± 2.7	0.70
Smokers, %	8.4%	6.8%	0.36
Physical fitness level, 1–10 scale	6.9 ± 1.8	6.1 ± 2.2	<0.0001

^a^ Probabilities of differences were determined by *t*-test or chi-square.

Table 2 illustrates demographic and dietary variables across categories of fruit and vegetable intakes. Subjects who consumed more fruits and vegetables per day were generally women, those with a higher physical fitness level, and those with higher meat consumption. Fruit and vegetable consumption were highly correlated. Table 3 shows the biomarker concentrations of subjects across categories of fruit and vegetable intakes. There was significant drift in several of the variables over the 12 weeks of the study. Higher intakes of both fruits and vegetables were associated with lower concentrations of CRP, IL-6, and TNF-α inflammatory markers. Fruit and vegetable intakes were also positively correlated with FRAP and ORAC antioxidant capacity indictors and negatively associated with F_2_-isoprostane levels, indicating higher antioxidant capacity and lower levels of oxidative damage.

Table 4 presents the relationship between combined F&V intake and inflammatory and oxidative status markers. This table describes correlations across categories for both age-gender and multivariate adjusted models. Among the inflammatory markers IL-6 and TNF-α were significantly lower across increasing categories of combined F&V intake. FRAP was higher and F_2_-isoprostanes were significantly lower across categories of F&V intake. ORAC’s *p*-value was attenuated in the multivariate model. All other markers, including CRP, were non-significant in these full models. 

**Figure 1:**
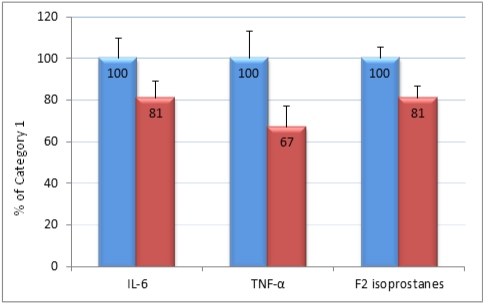
Comparison of category 3 to category 1 of combined fruit and vegetable intake for three biomarkers of inflammation and oxidative stress. For each marker the blue 100% bar represents the value of category 1 while the red bar represents category 3. The error bars are the 95% confidence intervals. The differences between category 1 and 3 for each marker are statistically significant at *p* ≤ 0.001.

Table 5 presents a subgroup analysis by gender. The F&V trend for IL-6 was only significant for women. TNF-α and F_2_-isoprostanes showed a significant interaction by gender. Figure 1 shows the significant lower markers of inflammation, IL-6 and TNF-α, and of oxidative damage, F_2_-isoprostanes, between category 1 of combined F&V intake, set at 100%, and category 3. The error bars are the confidence intervals for each category. The inter-category differences are statistically significant.

**Table 2:** Subject characteristics (mean ± standard deviation) by category of fruit and vegetable intake of community cohort.

**Categories of fruit intake**				
Variables	<2 servings daily	2 servings daily	>2 servings daily	*p* for trend ^a^
Category Number	*N* = 274	*N* = 361	*N* = 360	
Age, years	44.2 ± 14.9	46.1 ± 16.3	47.1 ± 17.1	0.13
Female	52%	62%	66%	0.0064
BMI, kg/m^2^	27.9 ± 5.8	26.9 ± 5.8	25.9 ± 5.0	<0.0001
Smokers	13%	7%	4%	0.0003
Physical fitness level, 1–10 scale	5.8 ± 2.1	6.4 ± 2.0	6.8 ± 2.0	<0.0001
Vegetable levels, 1–5 scale	1.5 ± 0.8	2.0 ± 0.8	2.8 ± 1.4	<0.0001
Red Meat levels, 1–5 scale	1.0 ± 0.8	1.3 ± 0.9	1.3 ± 0.9	<0.0001
**Categories of vegetable intake**				
Variables	<3 servings daily	3 servings daily	>3 servings daily	*p* for trend ^a^
Category Number	*N* = 292	*N* = 409	*N* = 301	
Age, years	44.7 ± 15.7	45.1 ± 16.6	48.3 ± 16.2	0.018
Female	55%	58%	69%	0.0048
BMI, kg/m^2^	27.1 ± 5.7	27.1 ± 5.6	26.2 ± 5.4	0.051
Smokers	9%	8%	5%	0.20
Physical fitness level, 1–10 scale	6.1 ± 2.0	6.2 ± 2.1	6.9 ± 2.0	<0.0001
Fruit levels,1–5 scale	1.4 ± 0.8	2.2 ± 0.9	3.0 ± 1.1	<0.0001
Red Meat levels, 1–5 scale	0.8 ± 0.8	1.4 ± 0.9	1.3 ± 0.9	<0.0001

^a^ Probabilities of trends were determined by generalized linear models or logistic regression adjusted for age and gender.

**Table 3:** Outcome characteristics (least square means (95% confidence interval)) by category of fruit and vegetable intake of community cohort.

**Categories of fruit intake**				
Variables	<2 servings daily	2 servings daily	>2 servings daily	*p* for trend ^a^
Category Number	*N* = 266	*N* = 357	*N* = 345	
CRP, mg/L	1.80 (1.55–2.08)	1.42 (1.26–1.62) *	1.16 (1.02–1.33) * ^#^	0.0032
IL-6, pg/mL ^&^	1.81 (1.69–1.94)	1.59 (1.50–1.69) *	1.34 (1.26–1.42) * ^#^	<0.0001
TNF-α, pg/mL ^&^	1.95 (1.79–2.13)	1.67 (1.54–1.80) *	1.46 (1.35–1.58) * ^#^	<0.0001
WBC, 10^9^/L	6.13 (5.94–6.33)	5.91 (5.75–6.08) *	5.69 (5.52–5.86) * ^#^	0.0066
MCP-1, pg/mL	165 (159–171)	161 (156–166)	163 (157–168)	0.63
GCSF, pg/mL ^&^	34 (32–35)	33 (31–34)	32 (30–33) *	0.35
IL-10, pg/mL	1.45 (1.33–1.59)	1.50 (1.34–1.67)	1.40 (1.22–1.61)	0.72
FRAP, µmol/L ^b^^&^	544 (528–560)	598 (584–612) *	601 (586–615) *	<0.0001
ORAC, µmol/L ^b^^&^	28.9 (28.3–29.6)	29.7 (29.1–30.4) *	30.1 (29.4–30.8) *	0.081
F_2_-isoprostanes, pg/mL	43.9 (42.3–45.4)	40.9 (39.6–42.1) *	36.8 (35.6–37.9) * ^#^	<0.0001
**Categories of vegetable intake**				
Variables	<3 servings daily	3 servings daily	>3 servings daily	*p* for trend ^a^
Category Number	*N* = 292	*N* = 409	*N* = 301	
CRP, mg/L	1.69 (1.47–1.95)	1.40 (1.24–1.58) *	1.20 (1.04–1.38) * ^#^	0.029
IL-6, pg/mL ^&^	1.73 (1.61–1.85)	1.56 (1.48–1.66) *	1.38 (1.29–1.48) * ^#^	<0.0013
TNF-α, pg/mL ^&^	2.22 (2.05–2.42)	1.56 (1.45–1.67) *	1.36 (1.25–1.48) * ^#^	<0.0001
WBC, 10^9^/L	5.98 (5.79–6.16)	5.94 (5.79–6.10)	5.75 (5.57–5.94) * ^#^	0.56
MCP-1, pg/mL	164 (158–170)	162 (157–167)	162 (156–168)	0.91
GCSF, pg/mL ^&^	33 (31–35)	33 (31–34)	32 (31–34)	0.87
IL-10, pg/mL	1.44 (1.33–1.57)	1.43 (1.27–1.61)	1.57 (1.34–1.84)	0.55
FRAP, µmol/L ^b^^&^	537 (521–552)	607 (594–620) *	598 (582–614) *	<0.0001
ORAC, µmol/L ^b^^&^	28.6 (28.0–29.2)	30.3 (29.7–30.9) *	29.9 (29.1–30.6) *	0.0009
F_2_-isoprostanes, pg/mL	42.3 (40.8–43.8)	40.1 (38.9–41.3) *	38.2 (36.9–39.6) * ^#^	0.0023

^a^ Probabilities of trends were determined by repeated measures generalized linear models adjusted for age and gender. Category values were determined by *post hoc* analysis of generalized linear models using the average values of the variables with Bonferroni’s adjustments. Statistics performed on log transformed values are presented as antilogs. Log transformed variables were CRP, IL-6, TNF-α, MCP-1, IL-10, and F_2_-isoprostanes. ^b^ FRAP is expressed as ascorbic acid equivalents in µmol/L, ORAC is expressed in trolox µmol/L. ^&^ Significant differences between the first and second measurement of these variables by paired *t*-test. * Significant difference with category 1. ^#^ Significant difference with category 2.

**Table 4:** Outcome characteristics (least square means (95% confidence interval)) by category of combined fruit and vegetable intake category in a community cohort.

**Category of combined fruit and vegetable intake from low to high**					
Marker	Category 1 *N* = 181	Category 2 *N* = 551	Category 3 *N* = 190	*p* for trend: age-gender model	*p* for trend: full model ^a^
CRP, mg/L	1.58 (1.31–1.90)	1.56 (1.36-–1.80)	1.43 (1.18–1.73)	0.0061	0.56
IL-6, pg/mL	1.77 (1.61–1.94)	1.69 (1.58–1.81)	1.46 (1.32–1.61) * ^#^	<0.0001	0.0073
TNF-α, pg/mL	2.06 (1.81–2.34)	1.73 (1.57–1.90) *	1.41 (1.23–1.60) * ^#^	<0.0001	<0.0001
MCP-1, pg/mL	6.44 (6.17–6.71)	6.43 (6.23–6.63)	6.30 (6.03–6.58)	0.62	0.58
IL-10, pg/mL	178 (158–197)	179 (164–194)	187 (167–207)	0.85	0.91
GCSF, pg/mL	35.2 (33.8–37.7)	35.5 (33.7–37.4)	34.7 (32.1–37.3)	0.22	0.78
WBC, 10^9^/L	1.43 (1.25–1.64)	1.52 (1.34–1.72)	1.70 (1.36–2.13)	0.77	0.33
FRAP, µmol/L ^b^	529 (504–553)	579 (561–597) *	602 (578–627) *	<0.0001	<0.0001
ORAC, µmol/L ^b^	28.7 (27.7–29.6)	29.4 (28.6–30.2)	30.3 (29.1–31.4) *	0.0047	0.047
F_2_-isoprostanes, pg/mL	48.2 (45.6–50.8)	44.7 (42.7–46.6) *	39.7 (37.0–42.4) * ^#^	<0.0001	<0.0001

^a^ Full model controlled for age, gender, BMI, smoking, physical fitness, and red meat intake. Statistics were performed as in Table 3. ^b^ FRAP is expressed as ascorbic acid equivalents in µmol/L, ORAC is expressed in trolox µmol/L. * Significant difference with category 1. ^#^ Significant difference with category 2.

**Table 5:** Subgroup analysis of outcome characteristics for men and women (least square mean (95% confidence interval)) by category of combined fruit and vegetable intake category in a community setting.

**Category of combined fruit and vegetable intake from low to high for men**					
Variables	Category 1	Category 2	Category 3	*p* for trend: full model ^a^	*p* for gender interaction
Category Number	*N* = 93	*N* = 221	*N* = 62		
CRP, mg/L	1.24 (0.97–1.59)	1.32 (1.08–1.61)	1.10 (0.81–1.48)	0.40	0.63
IL-6, pg/mL	1.78 (1.55–2.05)	1.77 (1.59–1.97)	1.69 (1.43–2.00)	0.81	0.33
TNF-α, pg/mL	2.09 (1.74–2.52)	1.60 (1.38–1.84) *	1.71 (1.36–2.14) *	0.018	0.0033
WBC, 10^9^/L	6.15 (5.78–6.53)	6.37 (6.08–6.66)	6.29 (5.84–6.73)	0.52	0.32
MCP-1, pg/mL	173 (162–185)	169 (161–179)	177 (163–192)	0.50	0.55
GCSF, pg/mL	31.0 (27.6–34.4)	34.7 (32.1–37.3) *	32.5 (28.5–36.6)	0.076	0.11
IL-10, pg/mL	1.56 (1.26–1.87)	1.74 (1.41–2.15)	2.14 (1.42–3.21) *	0.28	0.61
FRAP, µmol/L ^b^	1.56 (1.26–1.87)	1.74 (1.41–2.15)	2.14 (1.42–3.21) *	0.28	0.61
ORAC, µmol/L ^b^	28.0 (26.6–29.4)	29.3 (28.0–30.5)	29.5 (27.7–31.4) *	0.13	0.59
F_2_-isoprostanes, pg/mL	43.7 (40.6–47.0)	38.1 (35.9–40.2) *	36.0 (33.0–39.3) *	<0.0001	0.0064
**Category of combined fruit and vegetable intake from low to high for women**					
Variables	Category 1	Category 2	Category 3	*p* for trend: full model ^a^	*p* for gender interaction
Category Number	*N* = 91	*N* = 347	*N* = 132		
CRP, mg/L	1.99 (1.52–2.61)	1.86 (1.53–2.26)	1.80 (1.40–2.26)	0.81	0.63
IL-6, pg/mL	1.76 (1.55–2.00)	1.62 (1.48–1.78)	1.34 (1.20–1.51) * ^#^	0.0003	0.33
TNF-α, pg/mL	2.03 (1.70–2.43)	1.81 (1.60–2.06)	1.28 (1.09–1.50) * ^#^	<0.0001	0.0033
WBC, 10^9^/L	6.70 (6.32–7.08)	6.49 (6.22–6.76)	6.34 (5.99–6.69)	0.26	0.32
MCP-1, pg/mL	164 (151–177)	165 (156–174)	163 (152–175)	0.94	0.55
GCSF, pg/mL	39.5 (35.9–43.0)	36.8 (34.3–39.3)	36.9 (33.6–40.1)	0.29	0.11
IL-10, pg/mL	1.32 (1.10–1.59)	1.36 (1.16–1.58)	1.43 (1.10–1.87)	0.86	0.61
FRAP, µmol/L ^b^	467 (435–499)	534 (512–556) *	567 (539–596) * ^#^	<0.0001	0.61
ORAC, µmol/L ^b^	29.1 (27.7–30.4)	29.4 (28.4–30.5)	30.6 (29.1–32.1) * ^#^	0.15	0.59
F_2_-isoprostanes, pg/mL	43.6 (40.4–47.0)	44.4 (42.1–46.9)	38.7 (36.1–41.5) * ^#^	<0.0001	0.0064

^a^ Full model controlled for age, gender, BMI, smoking, physical fitness, and red meat intake. The interaction is based on the full model. Statistics were performed as in Table 3. ^b^ FRAP is expressed as ascorbic acid equivalents in µmol/L, ORAC is expressed in trolox µmol/L. * Significant difference with category 1. ^#^ Significant difference with category 2.

In this large community based study across a wide age and BMI range, self-reported combined F&V intake was correlated with biomarkers indicating lower inflammation and oxidative stress, and higher antioxidant power. With only limited attenuation after controlling for potentially confounding variables, cytokines IL-6 and TNF-α and oxidative damage marker F_2_-isoprostanes were lower over categories of combined F&V intake. In addition, FRAP and ORAC, both markers of potential protection from oxidative damage, were higher over the combined F&V intake categories. Thus, in this single observational study with a simple survey tool of food intakes and multiple biological markers of inflammatory and oxidative status, clear correlations are found between these combined food categories and both types of biological responses. Interestingly, CRP, a common marker of inflammation, failed to show a significant trend in this study.

Others have found similar, though not uniformly consistent results in more focused studies of inflammatory markers. Esmaillzadeh *et al.* found in a cross-sectional study of Tehrani female teachers that both F&V intake were associated with lower CRP [6]. Wannamethee *et al.*, in a study of older British men, found that fruit but not vegetable intake was associated with lower CRP [16]. In a study of adolescents, Holt *et al.* found fruit intake associated with lower CRP and vegetable intake with lower IL-6 [5]. TNF-α was not associated with either fruit or vegetable intake. In an interesting comparison of oxidant status of vegetarians and omnivores, Haldar *et al.* found no difference in most measured antioxidant concentrations, including FRAP, between the two dietary groups [17].

Controlled feeding experiments providing specific foods found equally mixed results. Duthie *et al.* found that cranberry juice supplementation for 2 weeks increased FRAP significantly, while Bub *et al.* found no effect on FRAP from 2 weeks supplementation with tomato juice, carrot juice, or spinach powder [18,19]. A diet containing 10 servings of F&V a day for 10 days increased ORAC, while 12 weeks of 500 mL of grape-orange-apricot drink did not [20,21].

Several cross-sectional studies have examined dietary patterns using factor analysis. In a report from the Nurses’ Health Study, a prudent dietary pattern, high in F&V, was inversely correlated with CRP but not IL-6 [22]. In a report from the Multi-Ethnic Study of Atherosclerosis (MESA) a dietary factor including fruits and leafy green vegetables was inversely correlated with both CRP and IL-6 concentrations, while a dietary factor rich in dark-yellow, cruciferous, and other vegetables was only correlated with IL-6 [23]. Among the Tehrani teachers mentioned above, Esmaillzadeh *et al.* showed that a healthy food pattern including F&V was inversely associated with CRP but not TNF-α or IL-6 [24].

Among the five inflammatory cytokines measured in our study, only two showed a significant inverse correlation with combined F&V intake. Does this suggest a weakness to the F&V-inflammation hypothesis? Other studies using multiple markers frequently find similar apparent inconsistencies, as noted above and in other similar studies [22,23,24,25]. As in our study, however, the inflammatory and oxidative damage markers are rarely positively correlated with F&V intake. In the present study, three markers, MCP-1, GCSF, and IL-10, have only recently been used in studies of food-induced inflammatory changes [8]. The approach of using multiple markers for conceptual endpoints such as inflammation or oxidative status is reaffirmed by our current study.

The ORAC and FRAP results, both measures of antioxidant capacity, gave slightly divergent results. This is not entirely unexpected since Cao and Prior have reported only a weak correlation between FRAP and ORAC in human serum samples [26]. FRAP measures the reductive capacity of the sample, thus inferring antioxidant capacity/potential [14]. The ORAC assay uses a free radical generating system to measure the antioxidant scavenging activity of the sample [15]. Based on the FRAP and F_2_-isoprostane data we conclude that F&V intake is correlated with higher antioxidant capacity and lower lipid peroxidation.

Five of the blood markers showed significant differences between the two measurements 12 weeks apart. This seasonal drift in is common in human studies and was partially ameliorated by the study design in which half of the subjects were recruited and studied from winter to spring and half were recruited and studied from summer to fall.

The limitations of the present study include the simplicity of the food frequency questionnaire. The F&V intakes were self-reported at a single point in time. Subjects in our study were highly educated, and predominantly White and non-smokers. Thus, applicability of our data to other populations may be limited.

A strength of this study included having data from two blood samples taken 12 weeks apart, thus reducing intra-subject variability. With 1000 subjects ranging widely in age, BMI, and chronic disease status, inferential credibility is increased. With multiple inflammatory cytokines and oxidant status markers, our understanding of the commonality of these two salutary pathways is also increased. Combining both F&V intakes helps focus our data on the public health recommendations of consuming more F&V of all kinds.

## 4. Conclusions

Within our population of 1000 community-dwelling adults, the upper category of combined F&V intake was related to lower plasma levels of two of five inflammatory cytokines and the oxidative stress biomarker F_2_-isoprostanes, and with elevated antioxidant capacity as represented by FRAP and ORAC. These findings are strengthened by the disparate characteristics of our subjects and statistical modeling that controlled for confounding due to age, BMI, gender, physical fitness level, smoking status, and red meat intake. These data support public health recommendations to increase F&V intake for the purpose of lowering chronic disease risk factors.

## 5. Implications

Public health recommendations widely support the increased consumption of fruits and vegetables over processed foods particularly high in refined grains and sugars and in fatty meats and dairy products. These research results support the recommendation of high fruit and vegetable intake, suggesting that such intake may be correlated with improved markers of vascular health and reduced risk of cardiovascular diseases.
